# IL-17A and IFN-γ Synergistically Induce RNase 7 Expression via STAT3 in Primary Keratinocytes

**DOI:** 10.1371/journal.pone.0059531

**Published:** 2013-03-21

**Authors:** Maren Simanski, Franziska Rademacher, Lena Schröder, Hanna Maria Schumacher, Regine Gläser, Jürgen Harder

**Affiliations:** Department of Dermatology, University Hospital Schleswig-Holstein, Kiel, Germany; Ludwig-Maximilian-University, Germany

## Abstract

Human keratinocytes produce several antimicrobial peptides and proteins (AMP) which contribute to the protection of human skin against infection. RNase 7 is a major AMP involved in cutaneous defense with a high expression in keratinocytes and a broad spectrum of antimicrobial activity. The cytokine IL-17A has been recently identified as a potent inducer of several AMP in keratinocytes. Since the role of IL-17A to induce RNase 7 expression is unknown we analyzed IL-17A alone and in combination with other cytokines to induce RNase 7 expression in keratinocytes. Whereas IL-17A alone only weakly induced RNase 7 expression, the synergistic combination of IL-17A and IFN-γ (IL-17A/IFN-γ) was identified as a potent inducer of RNase 7 expression. This combination was more effective in inducing RNase 7 than the combination of IL-17A/TNF-α, a combination previously identified as a strong inducer of psoriasis-related immune response genes including several AMP. IFN-γ and IL-17A both have been reported to activate the transcription factor STAT3 (Signal transducer and activator of transcription 3). Therefore we investigated the influence of STAT3 on the IL-17A/IFN-γ -mediated RNase 7 induction. The use of a STAT3 inhibitor as well as siRNA-mediated downregulation of STAT3 resulted in a diminished IL-17A/IFN-γ -mediated RNase 7 induction in keratinocytes indicating that STAT3 is involved in this process. Similarly as seen with RNase 7, treatment of keratinocytes with IL-17A/IFN-γ revealed also a synergistic induction of gene expression of the AMP human beta-defensin (hBD)-2 and -3 as well as the S100 protein psoriasin (S100A7) indicating that the combination of IL-17A/IFN-γ is a potent inducer of various AMP classes in general. This was also reflected by an increase of the *Staphylococcus aureus*-killing activity of IL-17A/IFN-γ -treated keratinocytes.

## Introduction

Human skin is able to protect itself from infections by the release of antimicrobial peptides and proteins (AMP). AMP represent a diverse group of small, mainly cationic endogenous proteins that exhibit a broad spectrum of antimicrobial activity [1]–[3]. Several *in vivo* studies have documented the capacity of AMP to protect the host against pathogenic microorganisms [4]–[7]. In addition to acting as antibiotic molecules several AMP display immunomodulatory activities such as induction of chemotaxis, cytokine release, angiogenesis and wound healing [8].

Important AMP that participate in cutaneous defense comprise the human beta-defensins (hBD)-2 and hBD-3 [9]–[12], the S100 protein psoriasin (S100A7c) [13], the cathelicidin LL-37 [5], [14], [15], the sweat gland-derived dermcidin [16] and the ribonuclease RNase 7 [17], [18]. RNase 7 is an important skin-derived AMP abundantly expressed in keratinocytes. Its broad-spectrum antimicrobial activity and its high expression in keratinocytes suggest that it may play an important role in cutaneous defense [17], [18]. Neutralization of RNase 7 by specific antibodies revealed that RNase 7 contributes to the capacity of human skin to control the growth of *S. aureus* [19]. In line with these data, Zanger and colleagues reported that travellers returning with *S. aureus* skin infections showed a significant lower constitutive RNase 7 gene expression than control subjects [20].

IL-17A is a cytokine with a central role in host defense [21]. For example, it plays an important role for protection against infection induced by intradermal injection of *S. aureus* [22]. In addition IL-17A has been reported to induce the expression of AMP in epithelial cells [23]–[25]. Because the capacity of IL-17A to induce RNase 7 has not yet been elucidated we sought to investigate the effects of IL-17A alone and in combination with other cytokines on RNase 7 expression in keratinocytes.

## Materials and Methods

### Culture of Epithelial Cells

Foreskin-derived primary keratinocytes pooled from different individuals were purchased from PromoCell (Heidelberg, Germany) and cultured in Epilife medium (Life Technologies, Carlsbad, USA) in a humidified atmosphere with 5% CO_2_. For stimulation experiments, cells were seeded in 12-well tissue culture plates (3.8 cm^2^/well, BD Biosciences) and used at 70–90% confluence. Cytokines used for stimulation were from PeproTech, Rocky Hill, NJ.

### RNA Isolation and cDNA Synthesis

After treatment, cells were washed with PBS and harvested using TRIzol reagent (Life Technologies) according to the supplier’s protocol. RNA quality and quantity were determined by photometry. Subsequently, 1 µg of total RNA was reverse transcribed to cDNA with oligo dT- primers and 50 Units Maxima Reverse Transcriptase (Thermo Fisher Scientific, Waltham, MA) according to the manufacturer’s protocol.

### Real-time PCR

Real-time PCR analyses were performed in a fluorescence temperature cycler (StepOne Plus, Life Technologies). cDNA corresponding to 10 ng RNA served as a template in a 10 µl reaction containing 0.5 µM of each primer and 1× SYBR Premix Ex Taq II Mix (TAKARA BIO, Otsu, Japan). Samples were loaded on 96 well plates and incubated in the fluorescence thermocycler (StepOne Plus) for an initial denaturation at 95°C for 30 s followed by 46 cycles, each cycle consisting of 95°C for 5 s and 60°C (touchdown of −1°C/cycle from 66°C to 60°C) for 30 s. At the end of each run, melting curve profiles were produced by an initial denaturation at 95°C for 15 s, cooling the sample to 60°C for 1 min and then heating slowly at 0.3°C/s up to 95°C with continuous measurement of fluorescence to confirm amplification of specific transcripts. Cycle-to-cycle fluorescence emission readings were monitored and analyzed using StepOne Software (Life Technologies).

The following intron spanning primers were used: RNase 7 5′- GGA GTC ACA GCA CGA AGA CCA -3′ (forward primer) and 5′- CAT GGC TGA GTT GCA TGC TTG A -3′ (reverse primer); hBD-2 5′- GCC TCT TCC AGG TGT TTT TG -3′ (forward primer) and 5′- GAG ACC ACA GGT GCC AAT TT -3′ (reverse primer); hBD-3 5′- AGC CTA GCA GCT ATG AGG ATC -3′ (forward primer) and 5′- CTT CGG CAG CAT TTT CGG CCA -3′ (reverse primer); psoriasin 5′- AGA CGT GAT GAC AAG ATT GAC -3′ (forward primer) and 5′- TGT CCT TTT TCT CAA AGA CGT C -3′ (reverse primer); ARP 5′- CAC CAT TGA AAT CCT GAG TGA TGT -3′ (forward primer) and 5′- TGA CCA GCC CAA AGG AGA AG -3′ (reverse primer). Standard curves were obtained for each primer set with serial dilutions of plasmids containing the amplification product. All quantifications were normalized to the housekeeping gene ARP (acidic ribosomal protein). Relative expression is given as a ratio between target gene expression and ARP expression.

### ELISA

Protein levels of RNase 7 were measured by ELISA as previously described [26]. The detection limit of the ELISA was 0.3 ng/ml.

### Antimicrobial Assays

Primary keratinocytes were cultured in 12-well plates in KGM2 medium (Promocell, Heidelberg, Germany) without antibiotics and were preincubated for 24 h with 1.7 mM CaCl_2_ prior to stimulation with the cytokine combination IL-17A/IFN-γ (each 20 ng/ml) or each cytokine alone (40 ng/ml) for 24 h. Subsequently, the culture supernatants were removed and cell lysates were prepared. Therefore, 500 µl water was added to each well, the cells were collected with a scraper and lysated by sonication. Debris was removed by centrifugation at 20,000×g at 4°C for 10 min. 40 µl of each lysate were transferred in a round bottom 96-well plate (Nunc, Thermo Fisher Scientific). Untreated cells served as control.

*S. aureus* strain SA113 *dltA* [27] was cultured at 37°C in TSB broth to an optical density at 600 nm (OD_600_) of 0.2, collected by centrifugation and resuspended in 10 mM sodium phosphate buffer, pH 7.4. 10 µl bacteria suspension was added to 40 µl lysate and incubated for 24 h at 37°C under humid conditions. The capacity of the keratinocyte lysates to inhibit bacterial growth was analyzed by measuring optical density at 600 nm in a tecan sunset reader (Crailsheim, Germany).

### Small Interfering RNA (siRNA)

STAT3 synthetic duplexed siRNA oligonucleotides were purchased from Qiagen. A validated nonsilencing siRNA was used as control (AllStars Negative Control siRNA, Qiagen). Primary keratinocytes were cultured in 12-well plates and used at 40–50% density on the day of transfection. Cells were transfected with 3 µl HiPerFect (Qiagen) and 30 nM siRNA in Epilife medium without antibiotics. After 18 h medium was changed to fresh Epilife medium. 30 h after transfection cells were used for stimulation with IL-17A/IFN-γ. The following primers were used to analyze the knock down efficiency of STAT3 gene expression by real-time PCR: 5′- CCT CTG CCG GAG AAA CAG -3′ (forward primer) and 5′- CTG CTC CAG GTA CCG TGT GT -3′ (reverse primer).

### STAT3 Inhibition Assay

Primary keratinocytes were treated at a confluence of 60–80% with 20 µg/ml of the synthetic STAT3 inhibitor VI (Merck, Darmstadt) for 6 h. Subsequently, cells were stimulated with IL-17A/IFN-γ for 16–20 h in the presence of 20 µg/ml STAT3 inhibitor. After stimulation gene expression of AMP was analyzed as described above.

## Results

### IL-17A/IFN-γ Synergistically Induce RNase 7 Protein Release in Primary Keratinocytes

**Figure 1:**
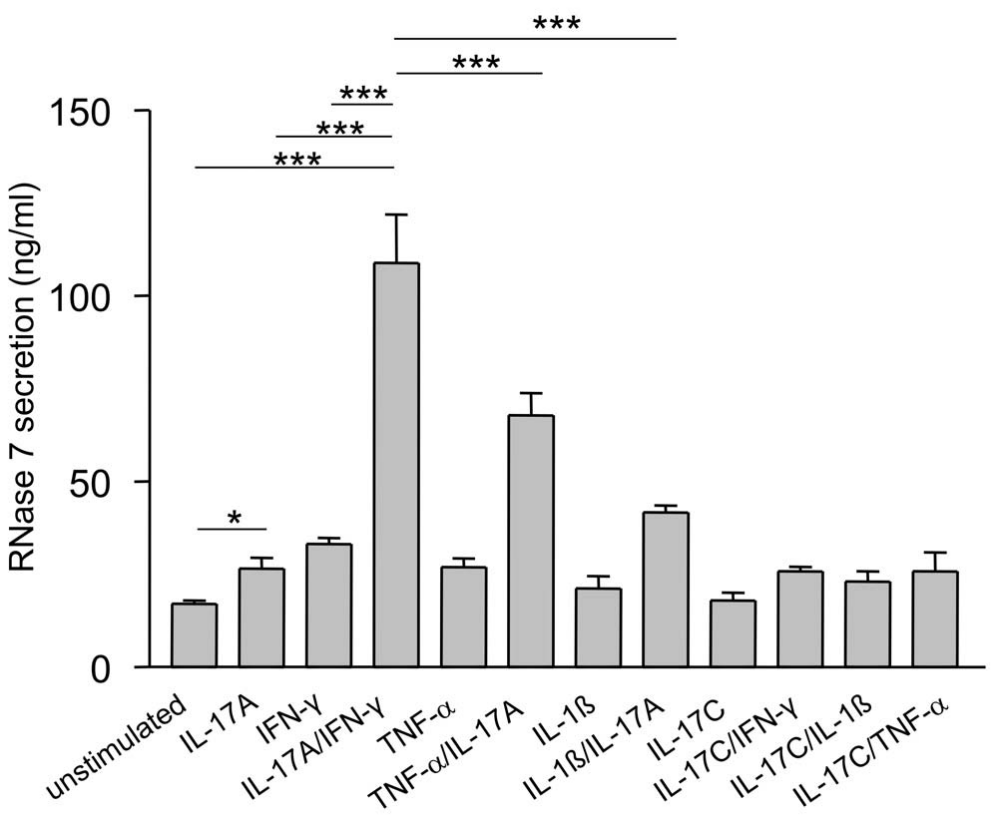
IFN-γ/IL-17 synergistically induce RNase 7 protein release in keratinocytes. Primary keratinocytes were stimulated with various cytokines (40 ng/ml) or cytokine combinations (each 20 ng/ml) for 48 h. Protein release of RNase 7 was analyzed by ELISA. Data are means ± SD (n = 3). Stimulation with IFN-γ/IL-17 induced RNase 7 release significantly (one-way ANOVA with Tukey’s test, ***p<0.001, *p<0.05).

To assess the capability of IL-17A to induce RNase 7 expression in keratinocytes we stimulated primary keratinocytes with 40 ng/ml IL-17A alone or in combination with IFN-γ, TNF-α, IL-1ß or IL-17C (each 20 ng/ml) for 48 h (Fig. 1). Secretion of RNase 7 was measured by ELISA analyses of the supernatants. Compared to an only moderate stimulation with IL-17A or IFN-γ alone, the combination of IL-17A and IFN-γ (IL-17A/IFN-γ) synergistically induced high levels of RNase 7 and was more effective in inducing RNase 7 than the combinations of IL-17A/IL-1ß or IL-17A/TNF-α. IL-17C alone was not able to induce secretion of RNase 7 and no synergistic action of IL-17C with other cytokines could be observed (Fig. 1).

### Dose-dependent Gene and Protein Expression of RNase 7 Upon Stimulation with IL-17A/IFN-γ

**Figure 2:**
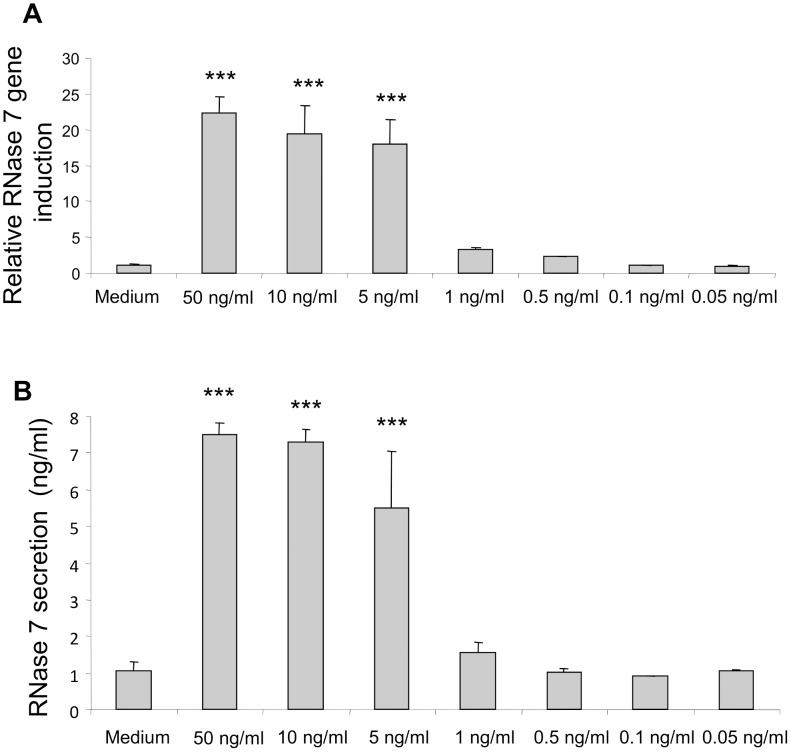
Dose-dependent gene and protein expression of RNase 7 upon stimulation with IFN-γ/IL-17.

Dose-response experiments showed that IL-17A/IFN-γ induced the gene expression of RNase 7 in a dose-dependent manner. Combined concentrations of IL-17A/IFN-γ as low as 1 ng/ml induced gene expression of RNase 7 in primary keratinocytes (Fig. 2a). To determine whether IL-17A/IFN-γ -induced RNase 7 gene expression correlates with protein expression we measured the secretion of RNase 7 using ELISA. As shown in Fig. 2b IL-17A/IFN-γ dose-dependently induced the release of RNase 7 indicating that protein expression correlates with gene expression.

### STAT3 Mediates the IL-17A/IFN-γ -induced Gene Expression of RNase 7

**Figure 3:**
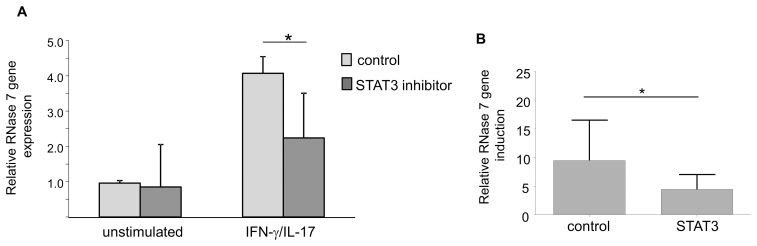
IFN-γ/IL-17A-induced RNase 7 gene expression is mediated by STAT3. (A) Primary keratinocytes were incubated with a STAT3 specific inhibitor for 1 h and subsequently stimulated with IFN-γ/IL-17A (each 20 ng/ml) for 18 h. Gene expression of RNase 7 was analyzed by real-time PCR. Keratinocytes without STAT3-inhibitor treatment served as control. Data are means ± SD of two independent experiments, each carried out in triplicates (*p<0.05, Student’s *t* test). (B) Primary keratinocytes were transfected with siRNA directed against STAT3 as well as a non-silencing control siRNA. 48 h after siRNA treatment cells were stimulated with IFN-γ/IL-17A (each 20 ng/ml) for 18 h. Gene expression of RNase 7 was analyzed by real-time PCR. Data are derived from four independent experiments each done in triplicate stimulations (*p<0.05, Student’s *t* test).

It has been shown that IFN-γ and IL-17A are able to activate the transcription factor STAT3 in keratinocytes [28], [29]. Therefore we investigated the influence of this transcription factor regarding the IL-17A/IFN-γ mediated induction of RNase 7. Treatment of the keratinocytes with a specific inhibitor of STAT3 signaling decreased the IL-17A/IFN-γ mediated gene induction of RNase 7 (Fig. 3a). To further evaluate the role of STAT3 for the IL-17A/IFN-γ -mediated RNase 7 induction we used siRNA directed against STAT3. The efficient knock-down of STAT3 mRNA was verified by real-time PCR revealing knock-down rates of more than 75% (data not shown). In concordance with the results obtained with the STAT3 inhibitor, downregulation of STAT3 expression using STAT3 specific siRNA significantly decreased the RNase 7 gene induction upon stimulation with IL-17A/IFN-γ (Fig. 3b).

### The Combination of IL-17A/IFN-γ Synergistically Induces the Gene Expression of the Antimicrobial Peptides hBD-2, hBD-3 and Psoriasin

**Figure 4:**
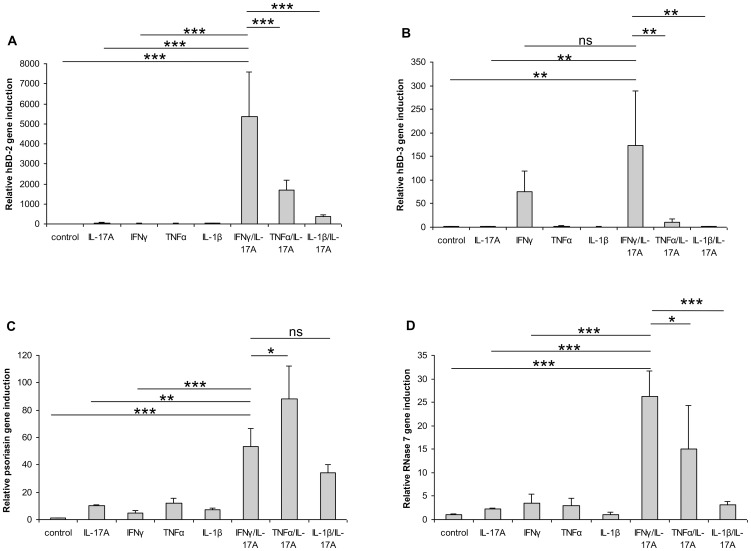
IFN-γ/IL-17 synergistically induce hBD-2, hBD-3, psoriasin and RNase 7 gene expression in keratinocytes. Primary keratinocytes were stimulated with various cytokines (40 ng/ml) or cytokine combinations (each 20 ng/ml) for 18 h. Gene expression of hBD-2 (A), hBD-3 (B), psoriasin (C) and RNase 7 (D) was analyzed by real-time PCR. Data are means ± SD (n = 3). Statistical analyses were done by one-way ANOVA with Tukey’s test (*p<0.05, **p<0.01, ***p<0.001, ns = not significant).

To analyze whether IL-17A/IFN-γ are able to synergistically induce other skin-relevant AMP, we analyzed the gene expression of hBD-2 and hBD-3 as well as psoriasin and compared it with RNase 7 gene expression. Gene expression of all four AMP was synergistically induced by the combination of IL-17A/IFN-γ (Fig. 4a–d). Similarly to RNase 7, the synergistic combination of IL-17A/IFN-γ was found to be the most potent cytokine combination to induce gene expression of hBD-2 (Fig. 4a) and hBD-3 (Fig. 4b). In the case of psoriasin, IL-17A/TNF-α was the most potent combination to synergistically induce gene expression followed by IL-17A/IFN-γ and 17A/IL-1ß (Fig. 4c).

### IL-17A/IFN-γ Enhances the Antimicrobial Activity of Keratinocytes

**Figure 5:**
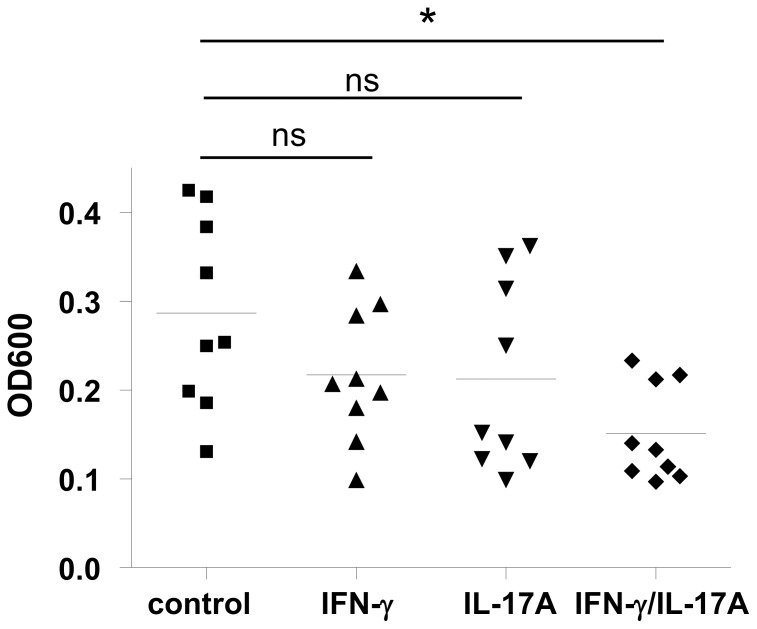
IFN-γ/IL-17 enhance the antimicrobial activity of primary keratinocytes. Primary keratinocytes were stimulated with IL-17A (40 ng/ml), IFN-γ (40 ng/ml) or IFN-γ/IL-17A (each 20 ng/ml) for 24 h. Cell lysates were incubated with *Staphylococcus aureus* and bacteria growth was determined by measuring optical density at 600 nm (OD_600_). Data are derived from three experiments each done in triplicates and statistical differences were analyzed using one-way ANOVA with Tukey’s test (*p<0.05, ns = not significant).

To analyze the functional relevance of the IL-17A/IFN-γ -mediated induction of AMP in keratinocytes we determined whether stimulation with IL-17A/IFN-γ enhanced the antimicrobial activity of keratinocytes. Therefore lysates of stimulated keratinocytes were coincubated with *S. aureus* SA113 *dltA* for 24 h and the optical density was measured to determine the capacity of the cell lysates to control *S. aureus* growth. Lysates of keratinocytes stimulated with IL-17A/IFN-γ significantly reduced the growth of *Staphylococcus aureus* as compared to keratinocytes stimulated with IFN-γ or IL-17A alone (Fig. 5).

## Discussion

Here we show that treatment of keratinocytes with IL-17A induced a moderate secretion of RNase 7. This effect was strongly enhanced by the combination of IL-17A/IFN-γ which synergistically induced RNase 7 gene expression and protein secretion in keratinocytes. Recently it has been reported that IL-17A in combination with TNF-α synergistically induced many psoriasis-related immune response genes including several cytokines and chemokines as well as AMP such as psoriasin and hBD-2 [30]. Our data revealed that the expression of RNase 7 was also synergistically induced by IL-17A/TNF-α. According to the published data we detected also a synergistically induced expression of hBD-2 by IL-17A/TNF-α. However, the induction of hBD-2 and RNase 7 by IL-17A/TNF-α was lower than the induction by IL-17A/IFN-γ. This indicates that the combination of IL-17A and IFN-γ is a powerful inducer of skin-derived AMP. In support of this hypothesis we detected also a strong induction of psoriasin and hBD-3 gene expression by this cytokine combination.

AMP are known to be overexpressed in the chronic inflammatory skin disease psoriasis vulgaris which may explain the low skin infection rate of patients suffering from this disease [31]. In this context it is discussed that the increased expression of AMP such as hBD-2 and hBD-3, which exhibit also chemotactic activities, may trigger psoriasis [32]. It is known that IFN-γ and IL-17A are associated with psoriasis and a synergistic activation of hBD-2 by these two cytokines has been reported [33]. These observations together with our results indicate that the synergistic activity of IFN-γ and IL-17A may contribute to the high levels of antimicrobial peptides such as RNase 7, psoriasin, hBD-2 and -3 in psoriasis [26], [34]. In addition, IL-17A plays an important role in cutaneous defense by the cooperation with other psoriasis-associated cytokines such as IL-22, TNF-α and IL-1 which have been implicated in a synergistic induction of AMP in keratinocytes [25], [30], [35]. Thus, it is likely that a cocktail of several cytokines contribute to the augmented expression of AMP in psoriasis.

In contrast to psoriasis, reduced levels of IFN-γ and IL-17A have been reported in atopic dermatitis [36]. The lower levels of the AMP-inducers IFN-γ and IL-17A may contribute to the observation that the induction of AMP in atopic dermatitis is less pronounced as compared to the AMP induction seen in psoriasis [37].

Using a synthetic STAT3 inhibitor as well as STAT3 siRNA we found that STAT3 is involved in the IL-17A/IFN-γ mediated induction of RNase 7. STAT3 mutations have been identified in patients with the hyper IgE-syndrome (HIES) [38]. Interestingly, these patients are characterized by recurrent skin infections caused by *Staphylococcus aureus.* It has been suggested that the HIES T-cell-derived cytokines fail to induce the production of anti-staphylococcal factors in keratinocytes [39]. There is evidence that RNase 7 contributes to cutaneous defense against *S. aureus* [19], [20]. Thus, it is intriguing to speculate that antimicrobial peptides that exhibit anti-staphylococcal activity and are regulated via STAT3 such as RNase 7 may be dysregulated in keratinocytes of HIES patients which may contribute to frequent *Staphylococcus aureus* infections. However, this hypothesis remains to be proven.

IL-17C is an epithelial derived cytokine recently implicated in the induction of epithelial defense responses such as AMP [40], [41]. However, our data revealed that neither IL-17C alone nor in synergy with IFN-γ or other cytokines induced the expression of RNase 7 in keratinocytes. In contrast, we observed a strong hBD-2 secretion upon IL-17C stimulation (not shown) which is line with the reported IL-17C-mediated induction of hBD-2 in keratinocytes [40]. This indicates that IL-17C in contrast to IL-17A plays no role in cutaneous defense to induce the expression of RNase 7. It is likely that the different influence of IL-17A and IL-17C on RNase 7 expression may be related to the fact that IL-17A signals via the receptors IL-17RA and IL-17RC whereas IL-17C signals via the 17RE–IL-17RA complex [41].

In summary, our study identified the synergistic activity of IL-17A and IFN-γ as a potent inducer of RNase 7 expression in keratinocytes. Besides RNase 7, other skin-derived AMP such as hBD-2, hBD-3 and psoriasin are strongly induced by IL-17A/IFN-γ indicating the powerful role of these cytokines to synergistically induce cutaneous innate defense. A better understanding of the regulation of AMP expression in keratinocytes may result in the development of novel therapeutic strategies enhancing the cutaneous innate defense system by induction of AMP.

## References

[1] Schauber J, Gallo RL (2008) Antimicrobial peptides and the skin immune defense system. J Allergy Clin Immunol 122: 261–266. 18439663 10.1016/j.jaci.2008.03.027 PMC2639779

[2] Selsted ME, Ouellette AJ (2005) Mammalian defensins in the antimicrobial immune response. Nat Immunol 6: 551–557. 15908936 10.1038/ni1206

[3] Harder J, Glaser R, Schröder JM (2007) Human antimicrobial proteins effectors of innate immunity. J Endotoxin Res 13: 317–338. 18182460 10.1177/0968051907088275

[4] Chromek M, Slamova Z, Bergman P, Kovacs L, Podracka L, et al (2006) The antimicrobial peptide cathelicidin protects the urinary tract against invasive bacterial infection. Nat Med 12: 636–641. 16751768 10.1038/nm1407

[5] Nizet V, Ohtake T, Lauth X, Trowbridge J, Rudisill J, et al (2001) Innate antimicrobial peptide protects the skin from invasive bacterial infection. Nature 414: 454–457. 11719807 10.1038/35106587

[6] Salzman NH, Ghosh D, Huttner KM, Paterson Y, Bevins CL (2003) Protection against enteric salmonellosis in transgenic mice expressing a human intestinal defensin. Nature 422: 522–526. 12660734 10.1038/nature01520

[7] Wilson CL, Ouellette AJ, Satchell DP, Ayabe T, Lopez-Boado YS, et al (1999) Regulation of intestinal alpha-defensin activation by the metalloproteinase matrilysin in innate host defense. Science 286: 113–117. 10506557 10.1126/science.286.5437.113

[8] Lai Y, Gallo RL (2009) AMPed up immunity: how antimicrobial peptides have multiple roles in immune defense. Trends Immunol 30: 131–141. 19217824 10.1016/j.it.2008.12.003 PMC2765035

[9] Pazgier M, Hoover DM, Yang D, Lu W, Lubkowski J (2006) Human beta-defensins. Cell Mol Life Sci 63: 1294–1313. 16710608 10.1007/s00018-005-5540-2 PMC11136124

[10] Harder J, Bartels J, Christophers E, Schröder JM (2001) Isolation and characterization of human beta -defensin-3, a novel human inducible peptide antibiotic. J Biol Chem 276: 5707–5713. 11085990 10.1074/jbc.M008557200

[11] Harder J, Bartels J, Christophers E, Schröder JM (1997) A peptide antibiotic from human skin. Nature 387: 861. 9202117 10.1038/43088

[12] Schröder JM (1999) Epithelial antimicrobial peptides: innate local host response elements. Cell Mol Life Sci 56: 32–46. 11213259 10.1007/s000180050004 PMC11147007

[13] Gläser R, Harder J, Lange H, Bartels J, Christophers E, et al (2005) Antimicrobial psoriasin (S100A7) protects human skin from Escherichia coli infection. Nat Immunol 6: 57–64. 15568027 10.1038/ni1142

[14] Gudmundsson GH, Agerberth B, Odeberg J, Bergman T, Olsson B, et al (1996) The human gene FALL39 and processing of the cathelin precursor to the antibacterial peptide LL-37 in granulocytes. Eur J Biochem 238: 325–332. 8681941 10.1111/j.1432-1033.1996.0325z.x

[15] Nizet V, Gallo RL (2003) Cathelicidins and innate defense against invasive bacterial infection. Scand J Infect Dis 35: 670–676. 14620153 10.1080/00365540310015629

[16] Schittek B, Hipfel R, Sauer B, Bauer J, Kalbacher H, et al (2001) Dermcidin: a novel human antibiotic peptide secreted by sweat glands. Nat Immunol 2: 1133–1137. 11694882 10.1038/ni732

[17] Koten B, Simanski M, Glaser R, Podschun R, Schröder JM, et al (2009) RNase 7 contributes to the cutaneous defense against Enterococcus faecium. PLoS One 4: e6424. 19641608 10.1371/journal.pone.0006424 PMC2712763

[18] Harder J, Schröder JM (2002) RNase 7, a novel innate immune defense antimicrobial protein of healthy human skin. J Biol Chem 277: 46779–46784. 12244054 10.1074/jbc.M207587200

[19] Simanski M, Dressel S, Gläser R, Harder J (2010) RNase 7 protects healthy skin from Staphylococcus aureus colonization. J Invest Dermatol 130: 2836–2838. 20668470 10.1038/jid.2010.217

[20] Zanger P, Holzer J, Schleucher R, Steffen H, Schittek B, et al (2009) Constitutive expression of the antimicrobial peptide RNase 7 is associated with Staphylococcus aureus infection of the skin. J Infect Dis 200: 1907–1915. 19919305 10.1086/648408

[21] Onishi RM, Gaffen SL (2010) Interleukin-17 and its target genes: mechanisms of interleukin-17 function in disease. Immunology 129: 311–321. 20409152 10.1111/j.1365-2567.2009.03240.x PMC2826676

[22] Cho JS, Pietras EM, Garcia NC, Ramos RI, Farzam DM, et al (2010) IL-17 is essential for host defense against cutaneous Staphylococcus aureus infection in mice. J Clin Invest 120: 1762–1773. 20364087 10.1172/JCI40891 PMC2860944

[23] Eyerich K, Pennino D, Scarponi C, Foerster S, Nasorri F, et al.. (2009) IL-17 in atopic eczema: linking allergen-specific adaptive and microbial-triggered innate immune response. J Allergy Clin Immunol 123: 59–66 e54. 10.1016/j.jaci.2008.10.031 19056110

[24] Kao CY, Chen Y, Thai P, Wachi S, Huang F, et al (2004) IL-17 markedly up-regulates beta-defensin-2 expression in human airway epithelium via JAK and NF-kappaB signaling pathways. J Immunol 173: 3482–3491. 15322213 10.4049/jimmunol.173.5.3482

[25] Liang SC, Tan XY, Luxenberg DP, Karim R, Dunussi-Joannopoulos K, et al (2006) Interleukin (IL)-22 and IL-17 are coexpressed by Th17 cells and cooperatively enhance expression of antimicrobial peptides. J Exp Med 203: 2271–2279. 16982811 10.1084/jem.20061308 PMC2118116

[26] Harder J, Dressel S, Wittersheim M, Cordes J, Meyer-Hoffert U, et al (2010) Enhanced expression and secretion of antimicrobial peptides in atopic dermatitis and after superficial skin injury. J Invest Dermatol 130: 1355–1364. 20107483 10.1038/jid.2009.432

[27] Peschel A, Otto M, Jack RW, Kalbacher H, Jung G, et al (1999) Inactivation of the dlt operon in Staphylococcus aureus confers sensitivity to defensins, protegrins, and other antimicrobial peptides. J Biol Chem 274: 8405–8410. 10085071 10.1074/jbc.274.13.8405

[28] Federici M, Giustizieri ML, Scarponi C, Girolomoni G, Albanesi C (2002) Impaired IFN-gamma-dependent inflammatory responses in human keratinocytes overexpressing the suppressor of cytokine signaling 1. J Immunol 169: 434–442. 12077274 10.4049/jimmunol.169.1.434

[29] Shi X, Jin L, Dang E, Chang T, Feng Z, et al (2011) IL-17A upregulates keratin 17 expression in keratinocytes through STAT1- and STAT3-dependent mechanisms. J Invest Dermatol 131: 2401–2408. 21796151 10.1038/jid.2011.222

[30] Chiricozzi A, Guttman-Yassky E, Suarez-Farinas M, Nograles KE, Tian S, et al (2011) Integrative responses to IL-17 and TNF-alpha in human keratinocytes account for key inflammatory pathogenic circuits in psoriasis. J Invest Dermatol 131: 677–687. 21085185 10.1038/jid.2010.340

[31] Henseler T, Christophers E (1995) Disease concomitance in psoriasis. J Am Acad Dermatol 32: 982–986. 7751469 10.1016/0190-9622(95)91336-x

[32] Hollox EJ, Huffmeier U, Zeeuwen PL, Palla R, Lascorz J, et al (2008) Psoriasis is associated with increased beta-defensin genomic copy number. Nat Genet 40: 23–25. 18059266 10.1038/ng.2007.48 PMC2447885

[33] Kryczek I, Bruce AT, Gudjonsson JE, Johnston A, Aphale A, et al (2008) Induction of IL-17+ T cell trafficking and development by IFN-gamma: mechanism and pathological relevance in psoriasis. J Immunol 181: 4733–4741. 18802076 10.4049/jimmunol.181.7.4733 PMC2677162

[34] Harder J, Schröder JM (2005) Psoriatic scales: a promising source for the isolation of human skin-derived antimicrobial proteins. J Leukoc Biol 77: 476–486. 15629886 10.1189/jlb.0704409

[35] Guilloteau K, Paris I, Pedretti N, Boniface K, Juchaux F, et al.. (2010) Skin Inflammation Induced by the Synergistic Action of IL-17A, IL-22, Oncostatin M, IL-1alpha, and TNF-alpha Recapitulates Some Features of Psoriasis. J Immunol. 10.4049/jimmunol.0902464 20335534

[36] Guttman-Yassky E, Lowes MA, Fuentes-Duculan J, Zaba LC, Cardinale I, et al (2008) Low expression of the IL-23/Th17 pathway in atopic dermatitis compared to psoriasis. J Immunol 181: 7420–7427. 18981165 10.4049/jimmunol.181.10.7420 PMC3470474

[37] de Jongh GJ, Zeeuwen PL, Kucharekova M, Pfundt R, van der Valk PG, et al (2005) High expression levels of keratinocyte antimicrobial proteins in psoriasis compared with atopic dermatitis. J Invest Dermatol 125: 1163–1173. 16354186 10.1111/j.0022-202X.2005.23935.x

[38] Minegishi Y, Saito M (2012) Cutaneous Manifestations of Hyper IgE Syndrome. Allergol Int 61: 191–196. 22441639 10.2332/allergolint.12-RAI-0423

[39] Minegishi Y, Saito M, Nagasawa M, Takada H, Hara T, et al (2009) Molecular explanation for the contradiction between systemic Th17 defect and localized bacterial infection in hyper-IgE syndrome. J Exp Med 206: 1291–1301. 19487419 10.1084/jem.20082767 PMC2715068

[40] Ramirez-Carrozzi V, Sambandam A, Luis E, Lin Z, Jeet S, et al (2011) IL-17C regulates the innate immune function of epithelial cells in an autocrine manner. Nat Immunol 12: 1159–1166. 21993848 10.1038/ni.2156

[41] Song X, Zhu S, Shi P, Liu Y, Shi Y, et al (2011) IL-17RE is the functional receptor for IL-17C and mediates mucosal immunity to infection with intestinal pathogens. Nat Immunol 12: 1151–1158. 21993849 10.1038/ni.2155

